# Complex Surgical Intervention for Small Bowel Obstruction Secondary to Metastatic Colorectal Cancer

**DOI:** 10.7759/cureus.45236

**Published:** 2023-09-14

**Authors:** Yesenia Brito, Shenika Vilton, Ana I Gonzalez, Scott Suddarth, Frederick Tiesenga

**Affiliations:** 1 Surgery, St. George's University School of Medicine, True Blue, GRD; 2 General Surgery, West Suburban Medical Center, Chicago, USA

**Keywords:** malignant bowel obstruction, obstruction, abdominal pain, mbo, sbo

## Abstract

Small bowel obstruction (SBO) refers to the inability of contents to pass through the lumen of the small intestine. This is a common surgical emergency in the United States. Although intra-abdominal adhesions are the predominant cause, SBO can occur secondarily to various etiologies, be it one cause or several. Management of SBO secondary to adhesions and metastasized rectal adenocarcinoma, complicated by pulmonary, hepatic, and ureteral disease, highlights the criticality of a multidisciplinary approach.

We present a case of a 59-year-old male with SBO secondary to rectal adenocarcinoma. Treatment included surgical resection, acute stabilization, referral for outpatient surgical follow-up, and oncologic management.

## Introduction

Small bowel obstruction (SBO) entails a disruption in the physiological movement of the gastrointestinal system. The most common cause of SBO in the United States is intraperitoneal adhesions, followed by mechanical obstructions and incarcerated herniations [1]. Tumors account for 20% of SBO cases [2]. Obstructions secondary to cholelithiasis or Crohn’s Disease are less frequently seen, the latter caused by stricture formation and chronic inflammatory processes accompanying the disease [3,4].

Signs of acute SBO include nausea, vomiting, abdominal pain, and obstipation [5]. The frequency and severity of these signs vary depending on the location and etiology of the obstruction. For example, if a patient complains of chronic hematochezia prior to the onset of acute abdominal pain, colon cancer should be considered among the differential causes of SBO, especially for those with advanced age, signs of anemia, positive family history of cancer, and lack of prior preventive colonoscopies. SBOs affect 28% of individuals with colorectal cancer and almost half of women with ovarian carcinomas [2].

Although patients with risk factors such as cancer or herniation can be suspected of SBO, physical exams and history are not enough to make a definitive diagnosis. Plain radiographs are used as a preliminary study to diagnose SBO and rule out the need for emergent surgical intervention [5]. Clinically stable patients can be further analyzed using abdominal and pelvic computed tomography (CT) scan to pinpoint the location and extent of the obstruction. Intraperitoneal adhesions, for example, can be classified as open or closed-loop obstructions. Open-loop refers to one point of obstruction in the bowel, while closed-loop describes blockage at two points [6]. Obstructions can be further classified as partial or complete, depending on the affected luminal surface area. Complete obstructions, such as closed-loop adhesions, have a higher incidence of complications ranging from reversible bowel ischemia to intestinal necrosis and sepsis.

Clinicians can look for multiple signs indicative of bowel ischemia on CT scan; these include thickening and enhancement of the bowel wall, prominent mesenteric venous supply, and peritoneal edema [6]. Free air under the diaphragm, also known as a pneumoperitoneum, indicates bowel perforation; this can be observed on plain radiographs but is more apparent on CT scan. Vascular compromise and bowel tears warrant immediate surgical intervention. On the other hand, hemodynamically stable patients can be treated conservatively by limiting oral intake, placing a nasogastric tube, and starting intravenous hydration using normal saline [3]. If conservative measures fail to resolve the SBO within 48 hours, surgery should be indicated for those who meet the criteria [2,5].

After resolution, the rate of recurrence of SBO is closely linked to the management. Patients who undergo surgical intervention tend to have a lower recurrence rate (13%) in comparison to those managed conservatively (21.3%) [7]. Conservative management is often a first-line treatment for open-loop adhesive SBO; however, a quarter of those subjected to nonoperative care fail to improve and ultimately require surgery [6]. Mechanical blockages, such as neoplasms, have better outcomes, and lower risks of symptom recurrence when removed [8]. Non-operative care for neoplastic obstructions has low rates of success [8], and although palliative surgery can have poor outcomes, the decision can be made along with the patient or health care proxy by taking into account benefits, risks, and post-operative quality of life.

## Case presentation

A 59-year-old male presented to the emergency department with a complaint of sudden-onset abdominal and suprapubic pain. The pain started twelve hours prior to the patient's arrival at the hospital and was progressively getting worse. Despite taking 1000 mg of acetaminophen, the pain did not improve. The patient also reported experiencing associated symptoms of nausea, vomiting, and diarrhea with streaks of blood. He denied dizziness, fever, chest pain, and shortness of breath.

The patient's past medical history was significant for advanced adenocarcinoma of the rectum with metastasis to the liver, which was surgically removed through an end colostomy in June 2022. A significant residual tumor attached to the sacrum, urinary bladder, and prostate area was also noted. Thereafter, the patient underwent palliative chemotherapy. Moreover, the patient has a history of diabetes mellitus, hypertension, and hyperlipidemia.

**Figure 1:**
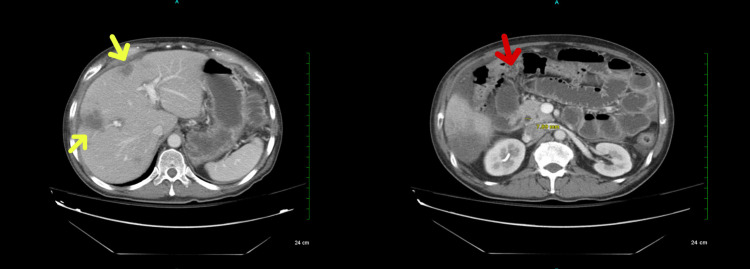
CT scan of abdomen and pelvis, showing numerous metastatic liver lesions (yellow arrows) and small bowel obstruction (red arrow).

On examination, the patient was hemodynamically stable. The vital signs were within normal limits. The abdomen was mildly distended, with tenderness to palpation. No guarding, masses, or organomegaly was present. A CT scan of the abdomen showed bowel obstruction and metastatic colorectal cancer (Figure 1).

**Figure 2:**
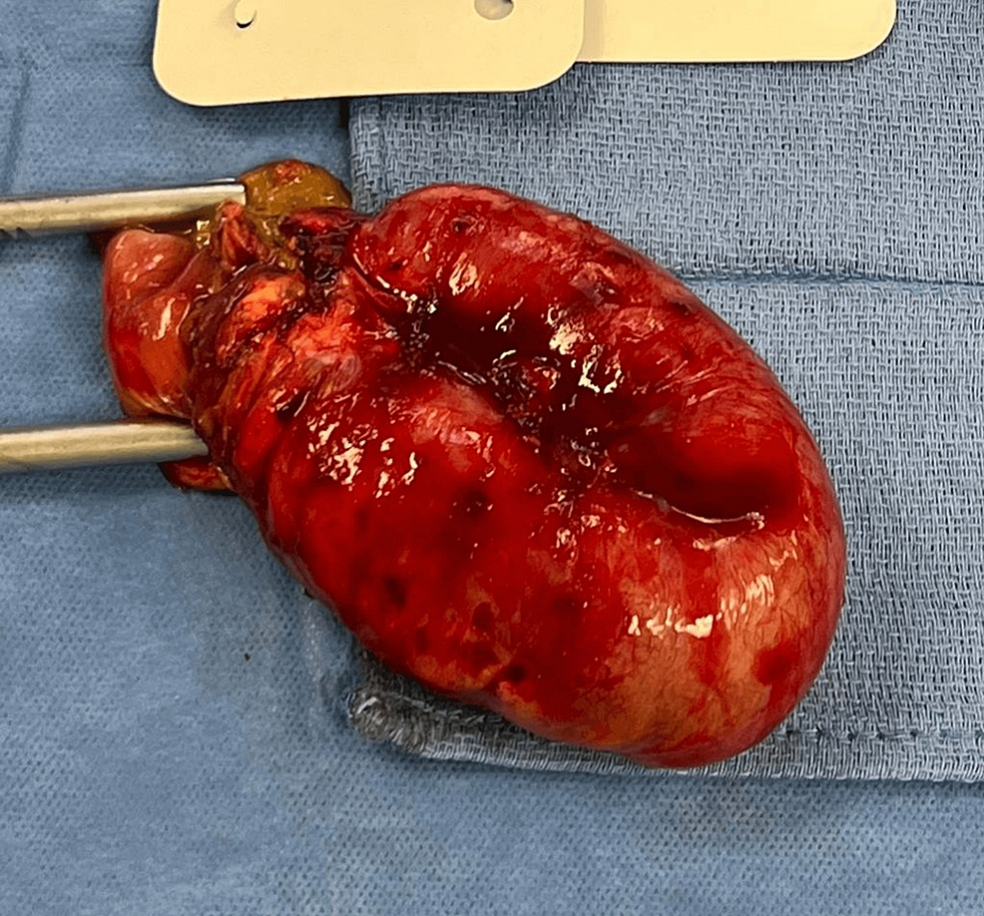
Resected small bowel.

The general surgical team was consulted and recommended the patient undergo exploratory laparotomy. During surgery, the abdomen was open through a midline incision. Upon entering the peritoneal cavity, a large amount of ascites was observed along with a massively dilated small bowel descending into the pelvis. Tumor burden was noted in the pelvis, where the small bowel was adhered to the tumor mass. The distal ileum was completely collapsed, causing a closed-loop obstruction in the pelvis. The affected small bowel was resected (Figure 2) and a new side-to-side anastomosis was created between the distal ileum and the proximal small bowel.

**Figure 3:**
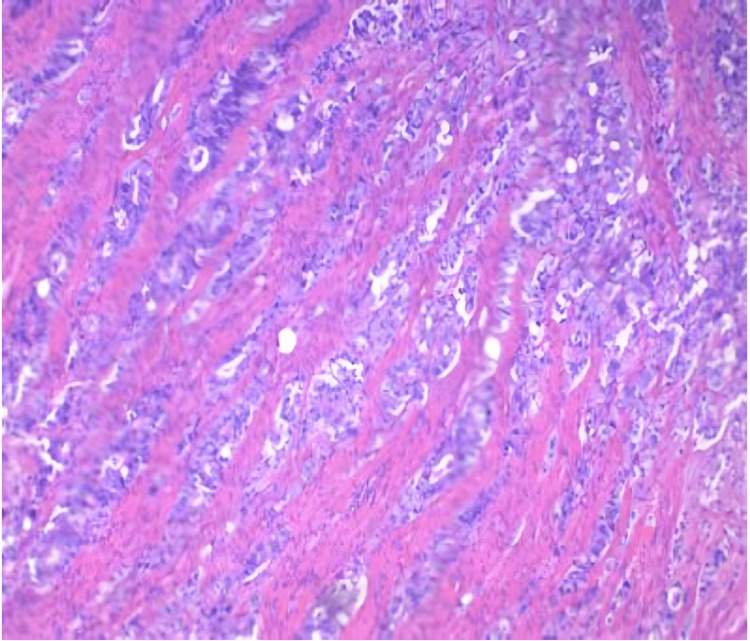
Hematoxylin & Eosin slide of small bowel tissue with serosal surface malignant glands.

Pathology report displayed small bowel tissue with serosal surface malignant glands (Figure 3). Immunohistochemistry shows the glands are positive for caudal-related homeobox transcription factor 2 (CDX2) and cytokeratin (CK) 20, and negative for CK7.

The patient’s postoperative course was uneventful. On postoperative day three, the diet was advanced to clear liquids, which was well tolerated. He denied any nausea, emesis, fever, or abdominal pain.

## Discussion

Small bowel obstruction (SBO) occurs when a blockage in the small intestine leads to the interruption of the intestine's normal flow. Symptoms of small bowel obstruction may include abdominal pain, distension, vomiting, constipation, and inability to pass gas [9]. The etiology of SBO can be broadly categorized into mechanical and non-mechanical factors. Mechanical causes include adhesions, hernias, tumors, volvulus, intussusception, strictures, and ingested foreign bodies to name a few [9,10]. Non-mechanical causes of SBO include paralytic ileus, inflammatory bowel disease, intestinal ischemia, radiation fibrosis, infections such as tuberculosis, and even endometriosis [9,10].

Annually, one in every 1,000 adults complains of acute abdomen during hospital admissions, which results in a diagnosis of small bowel obstruction. Elderly populations tend to be more susceptible to SBO due to the increased likelihood of past abdominal surgeries and inflammatory bowel diseases creating adhesions, inflammation, scarring, and strictures [11]. Other causes, such as hernias, tumors, volvulus, and intussusception are relatively less common but still contribute to the overall burden of SBO. Individuals who previously experienced SBO are at an increased risk of recurrence, especially if the initial cause, such as adhesions, has not been adequately addressed [11]. 

SBO is a potentially serious medical condition that can lead to various complications if not promptly diagnosed and treated. Complications can range from mild discomfort to life-threatening conditions. Some of the complications associated with small bowel obstruction include bowel ischemia, bowel gangrene, sepsis, electrolyte imbalance and dehydration due to vomiting, inability to eat or drink, fluid shifts, organ dysfunction, septic shock, scarring and adhesions, and delayed recovery [12,13]. 

The presented case illustrates a complex and challenging clinical scenario involving a patient with multiple coexisting medical conditions due to the progression and metastasis of adenocarcinoma. The patient’s medical conditions included, pulmonary nodules, cholelithiasis, hepatic lesions, rectosigmoid colonic mass with invasion into the urinary bladder and surrounding perirectal soft tissues, and hydroureteronephrosis. This presentation implied aggressive tumor behavior and raises concerns about the feasibility of surgical resection [14]. The involvement of several organs underscores the importance of multidisciplinary collaboration between urologists, surgeons, and oncologists in the management of this patient.

**Figure 4:**
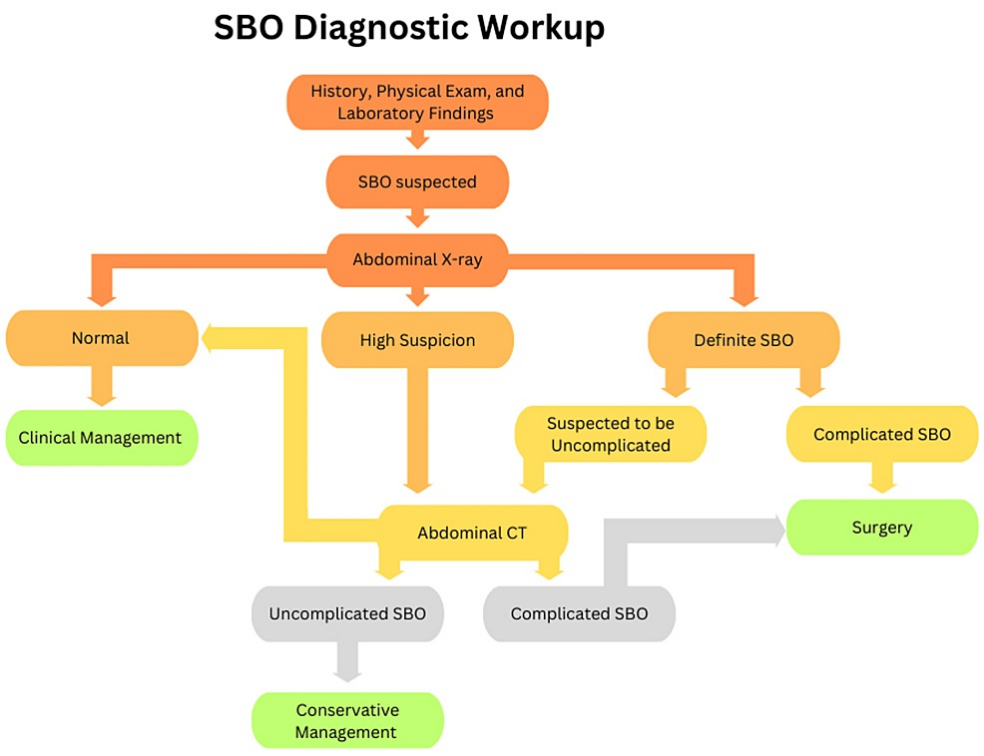
SBO diagnostic work-up. SBO: small bowel obstruction

Timely diagnosis and appropriate management are crucial for reducing complications and improving outcomes. Diagnostic management typically involves a combination of clinical assessment, imaging studies, and laboratory tests. The goal is to confirm the presence of obstruction, identify its location, determine the underlying cause, and assess any complications (Figure 4). 

Common protocols in conjugation with physical, history, and laboratory tests include abdominal X-ray and CT scan [10,11]. Treatment approaches can range from conservative measures to surgical intervention, depending on the severity of symptoms and complications. Management options commonly involve nasogastric decompression, intravenous fluids, pain management, exploratory laparotomy for severe obstructions, laparoscopic surgery, adhesiolysis, resection, stenting, and diverting stoma [10,11].

In recent years, small bowel follow-through (SBFT), which involves the administration of contrast material followed by X-ray imaging, has become valuable as it can predict which patients can be managed non-operatively and which will require surgery. This fluoroscope technology has been found effective in predicting surgery, reducing the need for surgery, and limiting inpatient stay duration in patients with adhesive SBO [15]. The contrast agent has a therapeutic osmotic effect that helps facilitate the movement of edema in the interstitial wall. This increases the pressure gradient across the obstruction, promoting bowel motility and leading to the resolution of the obstruction [16]. On the other hand, the available data does not offer conclusive support for the notion that SBFT has any effectiveness in addressing SBO caused by malignancy. There is insufficient evidence to support a reduction in the duration of obstruction and hospitalization time. The current data lacks sufficient evidence to indicate that contrast agent, along with abdominal radiographs, can reliably be used as a tool to predict the need for surgery [17].

When dealing with patients diagnosed with rectal adenocarcinoma and metastasis, the choice between exploratory laparotomy and laparoscopy is a critical decision that requires careful consideration. These two surgical approaches offer distinct advantages, each tailored to specific clinical scenarios. Exploratory laparotomy, characterized by a larger incision and comprehensive access, is an optimal choice when an exhaustive abdominal cavity exploration is imperative [18]. This approach facilitates a thorough assessment of the extent of disease, enables the execution of intricate surgical maneuvers, and proves beneficial when dealing with cases of complex metastatic lesions or potential bowel obstructions as in the case presented [18]. In contrast, laparoscopy, characterized by its minimally invasive nature, is a suitable option for stable patients who can tolerate a procedure with reduced postoperative discomfort, shorter hospitalization, and expedited recovery [18]. The ultimate decision hinges upon an intricate interplay of factors such as the patient's clinical condition, the expected invasiveness of procedures, the surgeon's proficiency, and the patient's preferences [18]. In the case of our patient, the risk for further metastasis warranted exploratory laparotomy over laparoscopy, allowing for a thorough assessment of disease spread, as well as necessary sampling and biopsy.

Mortality rates associated with SBO have improved over the years due to advancements in surgical techniques, imaging, and critical care management [19]. The mortality risk is generally associated with the underlying cause of obstruction and any complications that arise in overall diagnosis and treatment options [20].

## Conclusions

This case highlights the multifactorial nature of SBO. Although adhesions are the leading cause of SBO, it is important to follow an interdisciplinary approach. In cases such as this, with the involvement of adhesiolysis, tumor burden, and ostomy placement, especially given the extent of tissue invasion, short- and long-term goals of medical intervention warrant deliberation. 

Patient disposition at discharge following acute SBO resolution should be considered when deciding disease management. However, just as inpatient care is managed, a careful and attentive multidisciplinary follow-up is critical to optimize long-term healthcare priorities.
